# Phenotypic and Genotypic Traits of Vancomycin-Resistant Enterococci from Healthy Food-Producing Animals

**DOI:** 10.3390/microorganisms8020261

**Published:** 2020-02-15

**Authors:** Valerie Wist, Marina Morach, Marianne Schneeberger, Nicole Cernela, Marc J.A. Stevens, Katrin Zurfluh, Roger Stephan, Magdalena Nüesch-Inderbinen

**Affiliations:** Institute for Food Safety and Hygiene, Vetsuisse Faculty, University of Zurich, Winterthurerstrasse 272, 8057 Zurich, Switzerland; valerie.wist@uzh.ch (V.W.); mmorach@fsafety.uzh.ch (M.M.); marianne.schneeberger@vetbakt.uzh.ch (M.S.); n.cernela@access.uzh.ch (N.C.); katrin.zurfluh@uzh.ch (K.Z.); stephanr@fsafety.uzh.ch (R.S.)

**Keywords:** *Enterococcus faecium*, *E. faecalis*, *E. durans*, *vanA*, whole genome sequencing, Tn*1546*, food chain

## Abstract

Food-producing animals may be a reservoir of vancomycin-resistant enterococci (VRE), potentially posing a threat to animal and public health. The aims of this study were to estimate the faecal carriage of VRE among healthy cattle (*n* = 362), pigs (*n* = 350), sheep (*n* = 218), and poultry (*n* = 102 flocks) in Switzerland, and to characterise phenotypic and genotypic traits of the isolates. VRE were isolated from caecum content of six bovine, and 12 porcine samples respectively, and from pooled faecal matter collected from 16 poultry flock samples. All isolates harboured *vanA*. Three different types of Tn*1546*-like elements carrying the *vanA* operon were identified. Conjugal transfer of *vanA* to human *Enterococcus faecalis* strain JH2-2 was observed for porcine isolates only. Resistance to tetracycline and erythromycin was frequent among the isolates. Our data show that VRE harbouring *vanA* are present in healthy food-producing animals. The *vanA* gene from porcine isolates was transferable to other enterococci and these isolates might play a role in the dissemination of VRE in the food production chain.

## 1. Introduction

Antimicrobial resistance has now become a permanent aspect of human medicine with vancomycin-resistant enterococci (VRE) gaining importance as nosocomial pathogens worldwide [1]. The World Health Organization (WHO) ranks vancomycin-resistant *Enterococcus faecium* (VRE*fm*) as a pathogen of high priority in its global list of important antibiotic-resistant bacteria [2]. For European countries, the population-weighted mean percentage of resistance to vancomycin in invasive VRE*fm* increased from 10.5% in 2015 to 17.3% in 2018 [3]. By contrast, in *E. faecalis*, vancomycin resistance remains infrequent in Europe [3].

Nosocomial VRE*fm* may arise through independent events of introduction and subsequent dissemination within hospitals, but are also thought to generate within patients under antimicrobial therapy, most probably by the acquisition of resistance genes by means of horizontal gene transfer (HGT) [4,5,6,7]. One of the most important genetic determinants of vancomycin resistance is represented by the *vanA* gene cluster, which is organised as an operon consisting of *vanRSHAXYZ*, and is typically associated with transposons, such as Tn*1546* [8,9]. Tn*1546*-type transposons play a key role in the acquisition and dissemination of vancomycin resistance among enterococci [9,10]. Tn*1546* transposons vary structurally, because of point mutations, deletions, or the presence of insertion sequence (IS) elements [11,12]. These variations provide potential markers to type and trace the spread of *vanA* genes among enterococci isolated from different sources [13,14,15]. 

Most human clinical VRE*fm* strains belong to the *E. faecium* lineage designated Clade A1 [16,17]. This clade contains the vast majority of strains isolated from clinical settings, including isolates belonging to clonal complex (CC)17 [17,18], and to the recently emerged sequence types (STs)203 and ST796 [4,19,20,21]. Clade A2 contains strains that are predominantly associated with sporadic human infections and with livestock [18]. 

The proliferation of VRE in livestock in Europe is attributed to the past use of avoparcin, which was introduced in 1975 as a growth promoter, but which confers cross-resistance to vancomycin [22]. The EU ban on antimicrobial growth promoters enforced in 2006 (EC no. 1831/2003) lead to a decline of the prevalence of VRE among farm animals [22]. Nevertheless, VRE continues to be readily detected in samples from livestock when using selective media, and its persistence is suggested to be maintained by co-selection, i.e., the use of macrolides, tetracycline, or copper, or by the presence of plasmid addiction systems [22].

Accordingly, food-producing animals may be considered a reservoir of VRE that affects veterinary and human medicine, either by horizontal transfer of vancomycin resistance genes between animal and human adapted enterococci, or by clonal dissemination of resistant strains [22].

This study aimed to assess the occurrence of VRE among healthy cattle, pigs, poultry, and sheep at slaughter and to characterise and compare phenotypic and genotypic traits of the isolates from these different sources.

## 2. Materials and Methods 

### 2.1. Sampling and Bacterial Isolation

For slaughter cattle (*n* = 362), pigs (*n* = 350), and sheep (*n* = 218), swab samples were aseptically collected on 14 different days from caecal contents by cutting through the caecal wall with sterile scissors. Each caecum was swabbed once, avoiding an overload of faecal matter. Swabs were placed in sterile stomacher bags and transported to the laboratory. Animal and herd identification were collected along with each sample.

For poultry samples, faecal matter was collected on 9 different days at the entry of a poultry slaughterhouse from the crates of 102 poultry flocks (approximately 6000 chicken per flock). Pooled samples were placed in sterile bags and flock identification was noted for each sample. 

In the case of caecum samples, the excess lengths of the swabs were broken off, and 20 mL brain heart infusion (BHI) broth with 6.5% NaCl (Oxoid, Pratteln, Switzerland) was added to each bag. For poultry samples, broth was added directly to each bag. Samples were homogenised for 30–60′′ using a stomacher and incubated for 18–24h at 37 °C. From the pre-enrichment broth, one loopful was streaked onto VRE select agar (BioRad, Cressier, Switzerland) and incubated for 48 h at 37 °C.

From samples with presumptive enterococci positive colonies, colonies of different colony morphology were selected and purified on sheep blood agar (BioRad, Cressier, Switzerland). In case of unclear identity, isolates were tested for catalase activity and catalase negative isolates were further analysed. 

Species identification was performed by matrix-assisted laser desorption–ionization time of flight mass spectrometry (MALDI-TOF-MS, Bruker Daltonics, Billerica, MA, USA) using Compass FlexControl version 3.4 software with the Compass database version 4.1.80. All isolates were stored in 20% glycerol at –20 °C for further analysis.

### 2.2. Multiplex PCR Detection of vanA, vanB, vanC-1, and vanC-2/3 Genes 

Bacterial DNA was prepared by a standard lysis and boiling method [23]. Multiplex PCR targeting *vanA*, *vanB*, *vanC-1*, and *vanC-2/3* genes was carried out on all isolates using custom synthesised primers (Microsynth, Balgach, Switzerland) and conditions published previously [24]. Amplicons were visualised under ultraviolet light after electrophoresis in 1% agarose gel stained with ethidium bromide. *E. faecalis* ATCC51299 (*vanB*) and *Enterococcus casseliflavus* (*vanC*) [25] were used as positive controls. 

### 2.3. Antimicrobial Susceptibility Testing 

Determination of the minimal inhibitory concentrations (MICs) of vancomycin was performed using a gradient strip (Etest, Biomérieux, Geneva, Switzerland) according to the manufacturer’s instructions. Antimicrobial susceptibility profiling was performed using the Micronaut-S livestock 4 susceptibility plate (Merlin diagnostics GmbH, Berlin, Germany) with H-broth (Merlin diagnostics GmbH, Berlin, Germany). The MIC values were interpreted according to the Clinical and Laboratory Standards Institute (CLSI) susceptibility breakpoints, where available [26]. Antimicrobials with existing CLSI breakpoints included ampicillin (AMP), erythromycin (ERY), penicillin G (PEN), and tetracycline (TE).

### 2.4. Conjugation Experiments

Transfer experiments were performed using a modified solid mating protocol and *E. faecalis* JH2-2 (rifampicin resistant, vancomycin susceptible) as a recipient [27]. In brief, 40 µl volumes of overnight cultures of donor and recipient cells grown in BHI broth were mixed, concentrated by centrifugation, and resuspended in 20 µl of BHI broth. The mixture was dispensed onto BHI agar plates and incubated at 37 °C for 18–20 h. One loopful of cells were collected in 400 µl BHI broth. Serial dilutions were plated on BHI supplemented with 6 mg/L vancomycin and 50 mg/L rifampicin. The resulting transconjugants were purified and subject to identification by MALDI-TOF-MS.

Conjugation frequency was expressed as the number of transconjugants per recipient cell. Transconjugants were tested by singleplex PCR to confirm the presence of *vanA* using primers and conditions, described previously [24].

### 2.5. Whole Genome Sequencing

Bacterial cultures were grown overnight in 5 mL BHI with 6.5% NaCl. Chromosomal DNA was isolated from 1 mL overnight cultures using the DNeasy Blood and Tissue Kit (Qiagen, Hilden, Germany). Sequencing was done on an Illumina MiniSeq (Illumina, San Diego, CA, USA) and reads were assembled using Spades 3.12.0 [28] and Shovill [29], as described previously [30]. Species were identified using SpeciesFinder2.0 [31] available at https://cge.cbs.dtu.dk/services/SpeciesFinder/.

Draft genomes were annotated using Prokka and standard settings [32]. Resistance genes were identified using the resistance gene identifier (RGI) from the comprehensive antibiotic resistance database (CARD, available at https://card.mcmaster.ca/analyze/rgi) [33,34]. Searches were performed with standard setting and performed locally against the CARD database downloaded in September 2019.

Variations of the Tn*1546* element were identified based on what is considered the Tn*1546* prototype (GenBank M97297) [15,35]. Tn*1546* sequences were compared by using average nucleotide identity (ANI). The ANI was calculated according to Richter et al. [36], using the python script PyANI.py [37].

An ANI-base tree was constructed using an in-house R script. In short, the Euclidean distance in the relative-identity matrix produced by PyANI.py was calculated using the function “dist” from the package cluster [38] und subsequently clustered using the function “hclust”.

Core-genome multilocus sequence typing (cgMLST) was performed in the software package SeqSphere 4.1.9 (Ridom, Münster, Germany), the *Enterococcus faecium* MLST database (https://pubmlst.org/efaecium/), and the *Enterococcus faecalis* MLST database (https://pubmlst.org/efaecalis/), respectively, to determine sequence types (STs).

Alignments of the *vanA*-operon were visualised using CLC Main Workbench 8.1.3.

### 2.6. Accession Numbers

The whole genome shotgun sequences have been deposited at GenBank numbers VYUB00000000 to VYUX00000000.

## 3. Results

### 3.1. Prevalence of VRE among Healthy Food-Producing Animals

To determine the occurrence of VRE in slaughter animals in Switzerland, faecal samples collected during a 14-day trial were analysed. Overall, VRE were isolated from six (2%) of the cattle samples (all from cattle belonging to the same herd), 12 (3%) of the swine samples, and 16 (16%) of the poultry flocks. No VRE were isolated from sheep samples. A multiplex PCR targeting vancomycin resistance genes revealed the presence of *vanA* in all isolates. No *vanB*, *vanC-1*, or *vanC-2/3* genes were detected.

### 3.2. Antimicrobial Susceptibility Testing and Detection of Resistance Genes

A subset of 23 strains were selected for further analysis. To avoid sample clustering, one isolate from the positive cattle herd, and a selection of isolates from different pig herds were chosen. Strains included *E. faecalis* isolated from cattle (*n* = 1), *E. faecium* isolated from pigs (*n* = 6), *E. faecium* (*n* = 6) and Enterococcus *durans* (*n* = 10) isolated from poultry. Taken together, 12 *E. faecalis*, 10 *E. durans*, and one *E. faecalis* were analysed in this study.

All 23 strains exhibited MICs of vancomycin of > 32 µg/mL, confirming they were VRE (Table 1). 

Susceptibility profiling for additional antimicrobials with existing CLSI breakpoints showed that among the six *E. faecium* from pigs, the resistance phenotype PEN/TE, found in five isolates, represented the most common pattern (83%), followed by ERY/PEN/TE, detected in one isolate (17%).

Among the *E. faecium* from poultry, the ERY phenotype (determined in four isolates, 67%) predominated over the PEN phenotype (one isolate, 17%), and one isolate remained susceptible. 

For *E. durans*, the resistance pattern ERY/TE was observed in seven (70%) of the isolates, while TE was found in three (30%) of the isolates, respectively.

The *E. faecalis* isolated from cattle did not exhibit an additional resistance phenotype. 

Other antimicrobials included in the Micronaut-S livestock 4 susceptibility plate panel, to which enterococci are intrinsically resistant, or for which no breakpoints are available, had MIC values that are listed in Supplementary Table S1.

Regarding the resistance genotype, aminoglycoside resistance genes were detected among 22 (95.7%) of the 23 strains analysed, corresponding to all the *E. faecium* and *E. durans* strains and excluding the *E. faecalis* isolate. Tetracycline resistance genes were observed uniformly among *E. faecium* from pigs, in eight (80%) of the *E. durans,* but were absent in *E. faecium* for poultry and in the *E. faecalis* isolate (Table 1). Erythromycin genes were detected in eight (80%) of the *E. durans* strains. Notably, the lincosamide, streptogramin A, and pleuromutilin (LS_A_P) resistance gene *eat(A)_v_* was identified uniformly in *E. faecium* from pigs and from poultry (Table 1).

Genes potentially conferring metal-resistance including cadmium resistance genes *cadA* and *cadC*, the copper resistance gene *copZ*, the metal transport repressor gene *czrA*, mercury resistance genes *merA* and *merR*, and the putative zinc transporter gene *zosA*, were detected exclusively in isolates from pigs. 

### 3.3. Mating Experiments

To determine the risk of VRE spread, transfer experiments were performed. A subset of 16 strains, including one *E. faecalis* from cattle, six *E. faecium* from pigs, six *E. faecium* from poultry, and three *E. durans* from poultry were selected as donors for conjugative transfer to the recipient *E. faecalis* JH2-2. Vancomycin-resistant transconjugants were obtained from five donors, all of which were *E. faecium* isolates from pigs. Vancomycin resistance was transferred with frequencies of 1.7 × 10^−7^ (donor Sw245), 4.3 × 10^−7^ (donor Sw253), 5 × 10^−6^ (donor Sw290), 2 × 10^−6^ (Sw292), and 1.5 × 10^−5^ (Sw342), per recipient, respectively.

### 3.4. Characterisation of the Tn1546 Structures

**Figure 1:**
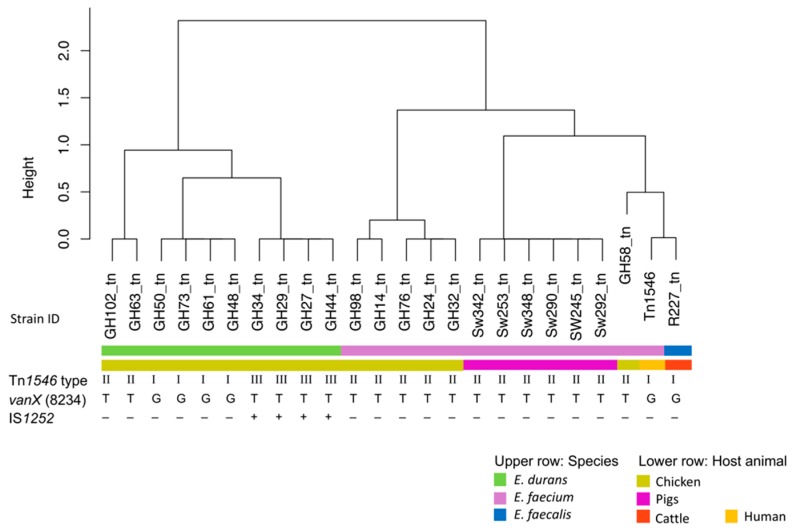
Average nucleotide identity (ANI) based cluster dendrogram showing three types of Tn*1546*-like elements carrying *vanA* operons identified in 23 *vanA-* type vancomycin-resistant enterococci from healthy food-producing animals. Type I corresponds to the prototype Tn*1546* (GenBank M97297) from human *E. faecium* B4147 [35]. Type II carries the G to T nucleotide point mutation at position 8234 in *vanX*. Tn*1546*-like element type III additionally carries an IS*1252* in the *orf*2-*van*R intergenic region.

Analysis of the Tn*1546* structures distinguished three different Tn*1546*-like types I-III, respectively (Table 1). A cluster dendrogram showing the Tn*1546* types of all the isolates analyzed in this study and the prototype Tn*1546* (M97297) is presented in Figure 1.

The structure of the *van* operon in type I was identical to the *van* operon prototype described previously (GenBank M97297), and included four *E. durans* isolates from poultry and the *E. faecalis* isolate from cattle (Table 1). The Tn*1546*-like type I elements detected in *E. durans* contained a topoisomerase gene downstream of *vanZ,* placing them in a highly similar but distinct cluster to the *E. faecalis* Tn*1546* (Figure 1).

**Figure 2:**
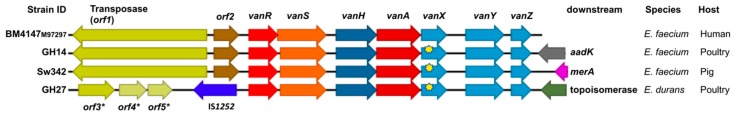
Linear maps of *vanA* encoding regions of the prototype Tn*1546* (GenBank M97297) from human *E. faecium* B4147 [35], and of vancomycin-resistant enterococci from healthy food-producing animals. Yellow stars indicate the G to T nucleotide point mutation at position 8234 in *vanX*; *aadK,* aminoglycoside 6-adenylyltransferase; *merA,* mercury resistance gene; *, putative open reading frames.

Type II Tn*1546*-like elements carried a G to T point mutation in *vanX* at position 8234 of the *van* operon and were identified in the six *E. faecium* from poultry, in the six *E. faecium* from pigs, and in two *E. durans* isolates from poultry (Table 1 and Figure 1). The *Tn1546*-like type II elements identified in *E. faecium* from poultry contained an *aadK* gene downstream of *vanZ*, whereas those found in pigs carried *merA* and those detected in *E. durans* contained a topoisomerase gene. Examples of type II Tn*1546*-like elements are shown in Figure 2. 

Finally, type III Tn*1546* was identical to the type II structure, but disrupted by IS*1252* in the *orf*2-*vanR* intergenic region. Type III elements were detected in four *E. durans* isolates from poultry and carried a topoisomerase gene located downstream of *vanZ* (Figure 2). 

### 3.5. Multilocus Sequence Typing

To determine the genetic diversity of the isolated, cgMLST was performed. Analysis of the 12 VRE*fm* revealed the occurrence of four different sequence types (STs). The most frequent ST was ST133, which was found in the six isolates from pigs. ST310 was identified in three, ST157 in two, and ST13 in one isolate from poultry, respectively (Table 1). MLST further assigned the *E. faecalis* from cattle to ST29 (Table 1).

## 4. Discussion

The use of antimicrobials as growth promoters was banned by law in Switzerland in 1999 [39]. During the decades that followed, VRE were detected at very low levels in the context of resistance monitoring of livestock, with only two porcine VRE*fc* isolated in 2009, and none from 2010 to 2012 [40]. However, between 2013 and 2016, one *E. faecalis* isolate from cattle, one *E. faecalis* from broilers, and two *E. faecium* isolates from fattening pigs were resistant to vancomycin, indicating that VRE are present in food animals in Switzerland [40,41].

The present study demonstrates the occurrence of *vanA*-type *E. faecalis*, *E. faecium*, and *E. durans* among Swiss cattle, pigs, and poultry flocks 20 years after the ban on avoparcin use. The persistence of VRE in the absence of an obvious selective pressure has been observed previously and is thought to be a consequence of co-selection through the therapeutic use of other antimicrobial agents, such as macrolides or tetracycline, and the use of metals, such as copper or zinc, as feed additives [42,43,44]. Accordingly, a high rate of phenotypic resistance to erythromycin and tetracycline was observed among the isolates in this study. Genetically, macrolide resistance encoded by *ermB* and tetracycline resistance encoded by *tet* genes, has been linked to the transposons of the Tn*1546* family that contain the *vanA* gene [42,45,46]. Correspondingly, *erm*(B), and *tet*(W) were frequently detected among the isolates. Furthermore, the *E. faecium* isolated from pigs in this study contained zinc and copper resistance genes, which are typical adaptations to the porcine environment [47].

Other resistance genes identified among the *E. faecium* isolates included the *aac(6*′*)-Ii* gene, which is ubiquitous in *E. faecium* and thought to contribute to intrinsic aminoglycoside resistance [48]. Similarly, *aac*(*6*′)-*Iid***,** which likewise is *E. durans* specific [49], was found uniformly among the *E. durans* isolates in this study. *E. faecalis* is intrinsically resistant to lincosamide, streptogramins A, and pleuromutilins (LS_A_P) due to the presence of *linA,* which was accordingly observed in the *E. faecalis* isolate R277 from cattle in this study. By contrast, in *E. faecium*, LS_A_P resistance is acquired by a C1349T point mutation in the Enterococcus ABC transporter gene *eat(*A*)* [50]. In human isolates, the mutated allelic variant *ea*t*(*A*)*_v_ has been reported in 23% of a collection of epidemiologically unrelated clinical isolates, including isolates corresponding to colonization or faecal carriage [50,51]. In the present study, *eat*(A)_v_ was identified uniformly in *E. faecium* from poultry and from pigs. To our knowledge, *eat(*A*)*_v_ has not been described previously among porcine and poultry associated *E. faecium* isolates. 

*VanA*-type resistance is generally mediated by Tn*1546*-like transposons that are frequently carried by self-transferable plasmids [9]. However, under the experimental conditions applied in this study, transfer of vancomycin resistance was obtained only from porcine donors. Our data confirm the possibility of *vanA* transfer from porcine *E. faecium* to human *E. faecalis*, as demonstrated previously in vitro and in vivo in the intestines of mice [52].

The Tn*1546* structures among the enterococci from this study were very similar. The majority contained the G8234T point mutation within *vanX,* which has been found in enterococcal isolates from healthy and hospitalised humans, in pig isolates, in food isolates, and in environmental enterococci [53,54,55,56]. This indicates that this transposon type is widely disseminated and shared between different enterococcal species and ecological niches. 

Many *vanA* type Tn*1546*-like structures contain insertion sequence (IS) elements that likely play a role in the evolution of vancomycin resistance [9]. IS*1216V* is one of the main IS elements frequently observed in the *orf2*-*vanR* and the *vanX*–*vanY* intergenic regions within Tn*1546* from different sources worldwide [14,53,57]. The absence of IS*1216V* within the Tn*1546* elements of the isolates from this study is characteristic for VRE*fm* identified previously in strains in Europe in the late 1990s and 2000s, indicating that the *vanA-*type resistance mechanism may be very conserved among livestock enterococci in Switzerland. The lack of the diversity observed in Tn*1546* structures, which is typical for human clinical isolates, suggests a limited sharing of resistance genes between livestock and human VRE, as observed previously for livestock and human enterococci isolates analysed in the United Kingdom [58].

Using cgMLST indicated that the *E. faecalis* and the *E. faecium* isolates belonged to STs typically identified among livestock-associated strains [55]. *E. faecium* isolated from pigs in this study were represented by a distinct population belonging to ST133. ST133 clusters within subgroup complex-5 (ST5), which contains STs that has been identified in pig hosts in Europe, including pigs from Denmark, Portugal, Spain, and Switzerland [54]. Further, ST5 has been found previously in healthy and in hospitalised patients in Denmark, Germany, and Portugal, notably however, unrelated to nosocomial spread [17,54]. These findings suggest a limited potential for transmission of VRE between humans and pigs. Similarly, *E. faecium* ST310, detected in three poultry isolates, is a poultry-adapted ST that is prevalent among broilers in Sweden [59,60], and *E. faecium* ST13 and ST157 have been reported in poultry in Sweden, Denmark, and Korea [55]. Taken together, MLST analysis did not reveal any close relationship to typical nosocomial strains belonging to CC17, or to recently emerged endemic strains ST203 and ST796. Likewise, *E. durans* is detected frequently in healthy poultry, but is reported only sporadically in human clinical infections [55]. *E. durans* of human and animal origin have been found to contain similar genetic arrangements of the *vanA* gene cluster, and it has been shown in vitro that *E. durans* transfers *vanA* to human clinical *E. faecium* at a high frequency [61,62]. Conversely, in our study, the *E. durans* did not result in transferability to *E. faecali*s, at least under the given experimental circumstances.

## 5. Conclusions

Our study provides further evidence of the occurrence of *vanA*-type VRE in livestock, including healthy cattle, pigs, and poultry. Our results suggest that porcine *E. faecium* may be prone to transfer *vanA* genes to human related *E. faecalis*. Furthermore, our data confirm previous studies that show that there is limited sharing of livestock-associated VRE strains with strains associated with sporadic human disease, and we did not identify any clones related to hospital-related outbreak strains, such as CC17.

## Figures and Tables

**Table 1:** Phenotypic and genotypic features of vancomycin-resistant *Enterococcus* spp. isolated from cattle, pigs, and poultry.

Host/Species	No. of Strains	Resistance Phenotype		Resistance Genotype		MLST
		MIC [µg/mL] Vancomycin	Additional Resistances^1^	Resistance Genes	Tn*1546* Type	
***Cattle***						
*E. faecalis*	1	≥128	–	*dfrE, emeA*, *efrA*, *efrB*, *lsaA*, *vanA*	I	29
***Pigs***						
*E. faecium*	1	≥256	PEN, ERY, TE	*aac(6*′*)-Ii, eat*(A)_v_, *cadA*, *cadC*, *copZ*, *czrA*, *merA*, *merR*, *tetW/N/W*, *vanA*, *zosA*	II	133
*E. faecium*	5	≥256	PEN, TE	*aac(6*′*)-Ii, eat*(A)_v_, *cadA*, *cadC*, *copZ*, *czrA*, *merA*, *merR*, *tetW/N/W*, *vanA*, *zosA*	II	133
***Poultry***						
*E. faecium*	1	≥256	ERY	*aac(6*′*)-Ii, aadK, eat*(A)_v_, *vanA*	II	13
*E. faecium*	1	≥256	PEN	*aac(6*′*)-Ii, aadK, eat*(A)_v_, *vanA*	II	157
*E. faecium*	1	≥256	–	*aac(6*′*)-Ii, aadK, eat*(A)_v_, *vanA*	II	157
*E. faecium*	3	≥256	ERY	*aac(6*′*)-Ii, aadK, eat*(A)_v_, *vanA*	II	310
*E. durans*	1	≥256	TE	*aac(6*′*)-Iid, tetW/N/W*, *vanA*	I	–
*E. durans*	2	≥256	ERY, TE	*aac(6*′*)-Iid, ermB*, *vanA*	I	–
*E. durans*	1	256	ERY, TE	*aac(6*′*)-Iid, ermB tetW/N/W*, *vanA*	I	–
*E. durans*	1	≥256	TE	*aac(6*′*)-Iid, ermB, tetW/N/W*, *vanA*	II	–
*E. durans*	1	≥256	ERY, TE	*aac(6*′*)-Iid, ermB tetW/N/W*, *vanA*	II	–
*E. durans*	3	≥256	ERY, TE	*aac(6*′*)-Iid, ermB tetW/N/W*, *vanA*	III	–
*E. durans*	1	≥256	TE	*aac(6*′*)-Iid, tetW/N/W*, *vanA*	III	–

Abbreviations: *aac(6*′*)-Ii* and *aac(6*′*)-Iid*: genes for aminoglycoside N-acetyltransferases; *aadK,* aminoglycoside 6-adenylyl-transferase; *cadA*, *cadC,* cadmium resistance genes; *cop*, copper resistance gene; *czrA*, metal transport repressor gene; *dfrE*, dihydrofolate reductase gene; *eat*(A)v, allelic variant of *eat*(A) gene for resistance to lincosamides, streptogramins A, and pleuromutilins (LSAP); *emeA*, enterococcal multidrug resistance efflux gene; *efrA*, *efrB*, ABC multidrug efflux pump genes; *ermB*, gene for 23S ribosomal RNA methyl-transferase; *lsaA*, active efflux ABC transporter gene for intrinsic LSAP resistance; *merA*, *merR*, mercury resistance genes; MIC, minimal inhibitory concentration; MLST, multilocus sequence type; *tetW/N/W*, mosaic tetracycline resistance gene and ribosomal protection protein; *vanA*, vancomycin resistance gene; *zosA*, zinc transporter gene.

## Supplementary Materials

The following are available online at https://www.mdpi.com/2076-2607/8/2/261/s1, Table S1: Minimal inhibitory concentrations (MICs) of antimicrobial agents for 23 vancomycin-resistant *Enterococcus* spp. isolated from healthy cattle, pigs, and poultry.
