# Correction: Wist et al. Phenotypic and Genotypic Traits of Vancomycin-Resistant Enterococci from Healthy Food-Producing Animals. *Microorganisms* 2020, *8*, 261

**DOI:** 10.3390/microorganisms9040847

**Published:** 2021-04-15

**Authors:** Valerie Wist, Marina Morach, Marianne Schneeberger, Nicole Cernela, Marc J. A. Stevens, Katrin Zurfluh, Roger Stephan, Magdalena Nüesch-Inderbinen

**Affiliations:** Institute for Food Safety and Hygiene, Vetsuisse Faculty, University of Zurich, Winterthurerstrasse 272, 8057 Zurich, Switzerland; valerie.wist@uzh.ch (V.W.); mmorach@fsafety.uzh.ch (M.M.); marianne.schneeberger@vetbakt.uzh.ch (M.S.); n.cernela@access.uzh.ch (N.C.); mstevens@fsafety.uzh.ch (M.J.A.S.); katrin.zurfluh@uzh.ch (K.Z.); stephanr@fsafety.uzh.ch (R.S.)

The authors wish to make the following correction to this paper [1].

After the publication of the manuscript, the authors recognized a discrepancy due to a nucleotide numbering difference between the prototype Tn*1546* (M97297) and the Tn*1546* elements sequenced in this study. Therefore, a point mutation within the *vanX* gene of six Tn*1546* elements described in this study was mistakenly reported.

We changed Table 1 and Figure 1 and Figure 2 and present the correct versions here.

The authors wish to point out the following:

The abstract should read: “two different types of Tn1*546*-like elements carrying the vanA operon were identified”.

The results Section 3.4. should read: “Analysis of the Tn*1546* structures distinguished two different Tn*1546*-like types I and II, respectively (Table 1).

The structure of the *van* operon in type I was identical to the *van* operon prototype described previously (GenBank M97297) and included six *E. durans* isolates from poultry, the *E. faecalis* isolate from cattle and *E. faecium* from pigs (Table 1). The Tn*1546*-like type I elements detected in *E. durans* contained a topoisomerase gene downstream of *vanZ*, placing them in a highly similar but distinct cluster to the *E. faecalis* Tn*1546* (Figure 1).

The Tn*1546*-like type I elements identified in *E. faecium* from poultry contained an *aadK* gene downstream of *vanZ*, whereas those found in pigs carried *merA*. Examples of the Tn*1546*-like elements are shown in Figure 2.

Finally, type II Tn*1546* was identical to the type I structure, but disrupted by IS*1252* in the *orf2*-*vanR* intergenic region. Type II elements were detected in four *E. durans* isolates from poultry and carried a topoisomerase gene located downstream of *vanZ* (Figure 2)”.

The discussion should read: “The majority corresponded to the prototype Tn*1546*, which has been found in enterococcal isolates from healthy and hospitalised humans, in pig isolates, in food isolates, and in environmental enterococci [53–56]”.

The authors would like to apologize for any inconvenience caused to the readers by these changes.

## Figures and Tables

**Figure 1:**
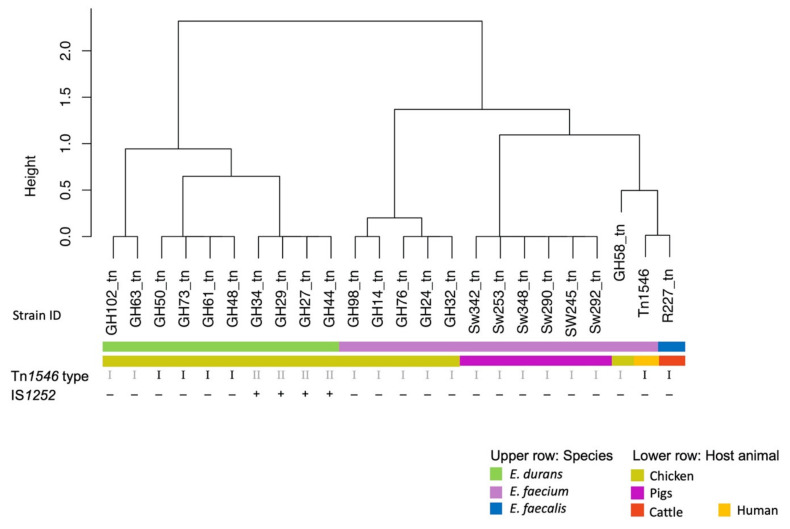
Average nucleotide identity (ANI) based cluster dendrogram showing three types of Tn*1546*-like elements carrying *vanA* operons identified in 23 *vanA*- type vancomycin-resistant enterococci from healthy food-producing animals. Type I corresponds to the prototype Tn*1546* (GenBank M97297) from human *E. faecium* B4147 [35]. Type II additionally carries an IS*1252* in the *orf2*-*vanR* intergenic region. Changed information is highlighted in grey.

**Figure 2:**
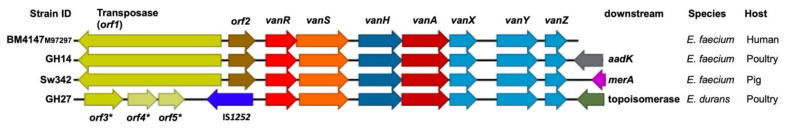
Linear maps of *vanA* encoding regions of the prototype Tn*1546* (GenBank M97297) from human *E. faecium* B4147 [35], and of vancomycin-resistant enterococci from healthy food-producing animals. *aadK*, aminoglycoside 6-adenylyltransferase; *merA*, mercury resistance gene; *, putative open reading frames.

**Table 1:** Phenotypic and genotypic features of vancomycin-resistant *Enterococcus* spp. isolated from cattle, pigs, and poultry. Changed information is highlighted in grey.

Host/Species	No. of Strains	Resistance Phenotype		Resistance Genotype		MLST
		MIC [µg/mL] Vancomycin	Additional Resistances	Resistance Genes	Tn*1546* Type	
***Cattle***						
*E. faecalis*	1	≥128	–	*dfrE, emeA*, *efrA*, *efrB*, *lsaA*, *vanA*	I	29
***Pigs***						
*E. faecium*	1	≥256	PEN, ERY, TE	*aac(6*′*)-Ii*, *eat*(A)_v_, *cadA*, *cadC*, *copZ*, *czrA*, *merA*, *merR*, *tetW/N/W*, *vanA*, *zosA*	I	133
*E. faecium*	5	≥256	PEN, TE	*aac(6*′*)-Ii*, *eat*(A)_v_, *cadA*, *cadC*, *copZ*, *czrA*, *merA*, *merR*, *tetW/N/W*, *vanA*, *zosA*	I	133
***Poultry***						
*E. faecium*	1	≥256	ERY	*aac(6*′*)-Ii*, *aadK*, *eat*(A)_v_, *vanA*	I	13
*E. faecium*	1	≥256	PEN	*aac(6*′*)-Ii*, *aadK*, *eat*(A)_v_, *vanA*	I	157
*E. faecium*	1	≥256	–	*aac(6*′*)-Ii*, *aadK*, *eat*(A)_v_, *vanA*	I	157
*E. faecium*	3	≥256	ERY	*aac(6*′*)-Ii*, *aadK*, *eat*(A)_v_, *vanA*	I	310
*E. durans*	1	≥256	TE	*aac(6*′*)-Iid*, *tetW/N/W*, *vanA*	I	–
*E. durans*	2	≥256	ERY, TE	*aac(6*′*)-Iid*, *ermB*, *vanA*	I	–
*E. durans*	1	256	ERY, TE	*aac(6*′*)-Iid*, *ermB tetW/N/W*, *vanA*	I	–
*E. durans*	1	≥256	TE	*aac(6*′*)-Iid*, *ermB*, *tetW/N/W*, *vanA*	I	–
*E. durans*	1	≥256	ERY, TE	*aac(6*′*)-Iid*, *ermB tetW/N/W*, *vanA*	I	–
*E. durans*	3	≥256	ERY, TE	*aac(6*′*)-Iid*, *ermB tetW/N/W*, *vanA*	II	–
*E. durans*	1	≥256	TE	*aac(6*′*)-Iid*, *tetW/N/W*, *vanA*	II	–

Abbreviations: *aac(6′)-Ii and aac(6′)-Iid*: genes for aminoglycoside N-acetyltransferases; *aadK*, aminoglycoside 6-adenylyl-transferase; *cadA*, *cadC*, cadmium resistance genes; *cop*, copper resistance gene; *czrA*, metal transport repressor gene; *dfrE*, dihydrofolate reductase gene; *eat(A)v*, allelic variant of *eat(A)* gene for resistance to lincosamides, streptogramins A, and pleuromutilins (LSAP); *emeA*, enterococcal multidrug resistance efflux gene; *efrA*, *efrB*, *ABC* multidrug efflux pump genes; *ermB*, gene for 23S ribosomal RNA methyl-transferase; ERY: erythromycin; *lsaA*, active efflux ABC transporter gene for intrinsic LSAP resistance; *merA*, *merR*, mercury resistance genes; MIC, minimal inhibitory concentration; MLST, multilocus sequence type; PEN: penicillin; TET: tetracyclin; *tetW/N/W*, mosaic tetracycline resistance gene and ribosomal protection protein; *vanA*, vancomycin resistance gene; *zosA*, zinc transporter gene.
